# Supplementary material for: HELP: A computational framework for labelling and predicting human common and context-specific essential genes
Source: PLoS Comput Biol. 2024 Sep 27;20(9):e1012076. doi: 10.1371/journal.pcbi.1012076 (PMC11463781; doi:10.1371/journal.pcbi.1012076)
Supplement: S1 Text — (PDF) [file pcbi.1012076.s001.pdf]

HELP: A computational framework for labelling and predicting human common and context-specific essential genes

Ilaria Granata[1*], Lucia Maddalena[1], Mario Manzo[2], Mario Rosario Guarracino[3,4], Maurizio Giordano[1]

Supplementary Figures and Tables

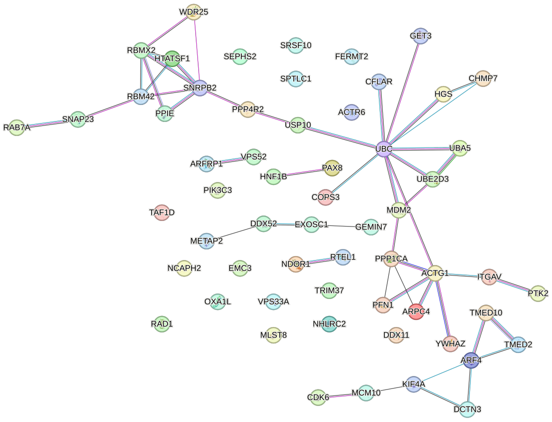

Category	Term ID	Term description	Count in network	FDR
GO Component	GO:0043231	Intracellular membrane-bounded organelle	55/12149	5.73E-05
GO Process	GO:0008152	Metabolic process	40/7988	0.0151
GO Process	GO:0071840	Cellular component organization or biogenesis	38/5639	9.22E-05
GO Process	GO:0008104	Protein localization	19/1943	0.0045
GO Process	GO:0015031	Protein transport	15/1180	0.0035
UniProt Keywords	KW-0597	Phosphoprotein	41/8122	0.0026
Reactome	HSA-162582	Signal Transduction	18/2540	0.0233

(A)

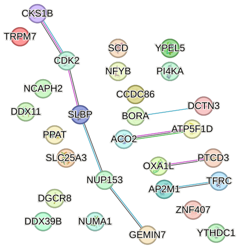

Category	Term ID	Term description	Count in network	FDR
GO Component	GO:0070013	Intracellular organelle lumen	20/5660	0.0075
UniProt Keywords	KW-0597	Phosphoprotein	22/8122	0.0219
Reactome	HSA-69278	Cell Cycle, Mitotic	7/526	0.0167

(B)

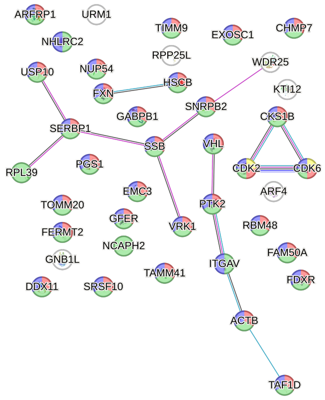

Category	Term ID	Term description	Count in network	FDR
GO Component	GO:0043229	Intracellular organelle	35/9609	0.0028
GO Component	GO:0043227	Membrane-bounded organelle	33/9083	0.0041
GO Component	GO:0043231	Intracellular membrane-bounded organelle	31/8162	0.0041
GO Component	GO:0097128	Cyclin D1-CDK4 complex	2/4	0.0206

(C)

Fig A. ucsEGs PPI enrichment. PPI networks built through STRING [1] using the ucsEGs computed for Kidney (A), Lung (B) and Brain (C). The nodes are coloured according to the enriched terms shown in the associated tables. The significant (False Discovery Rate, FDR $<$ 0.05) non-redundant terms were ranked by the number of enriching genes (Count in the network: no. of enriching genes/no. of genes annotated for the term). The edges were built with all the STRING information except "Text mining".

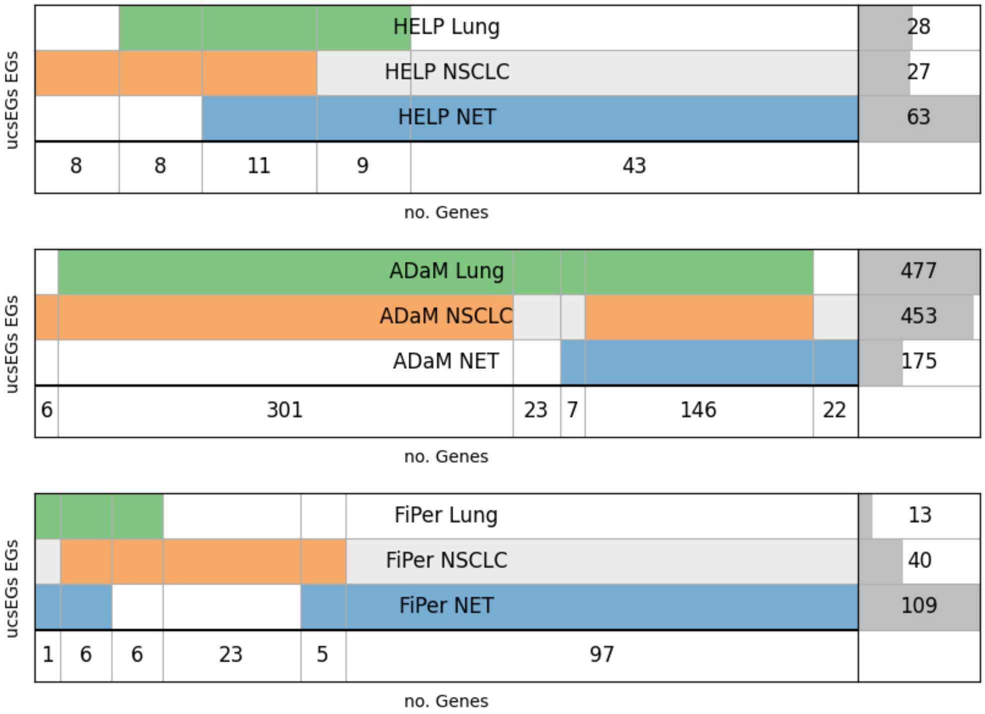

Fig B. Disease-specific ucsEGs. Diagram representing disease-specific (Non-Small-Cell Lung Cancer NSCLC and Lung Neuroendocrine Tumour NET) and lung ucsEGs intersections by ADaM, FiPer, and HELP labelling. Each row represents the set of ucsEGs for each labelling. The last row reports the number of genes resulting from the intersections. The last column on the right indicates the number of ucsEGs for each set, with the dark grey shadow representing the corresponding histogram.

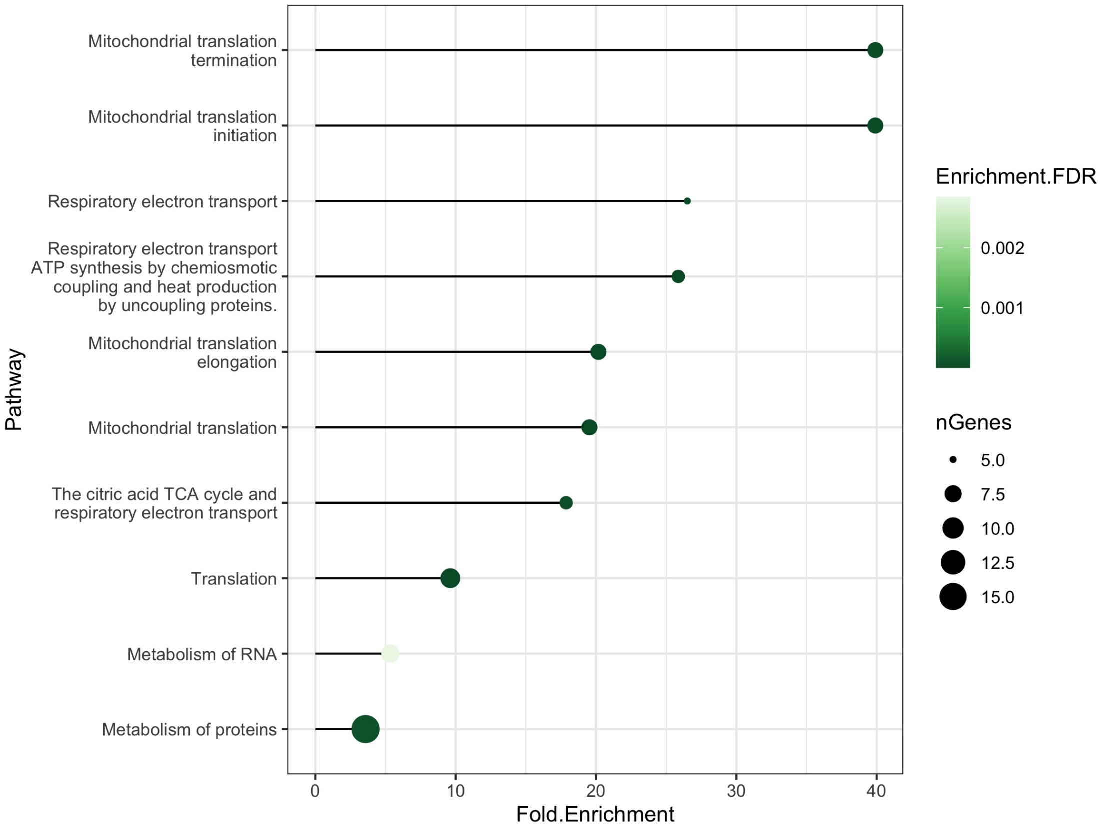

Fig C. Reactome pathway enrichment of lung NET-specific EGs. The significantly enriched pathways are shown on the y axis; the color bar indicates the significance in terms of False Discovery Rate (FDR)-adjusted p-value, while the dot size indicates the number of genes in the input set found in the pathway. On the x axis the Fold Enrichment, namely the percentage of genes in the input list annotated in a pathway divided by the corresponding percentage in the background human genes.

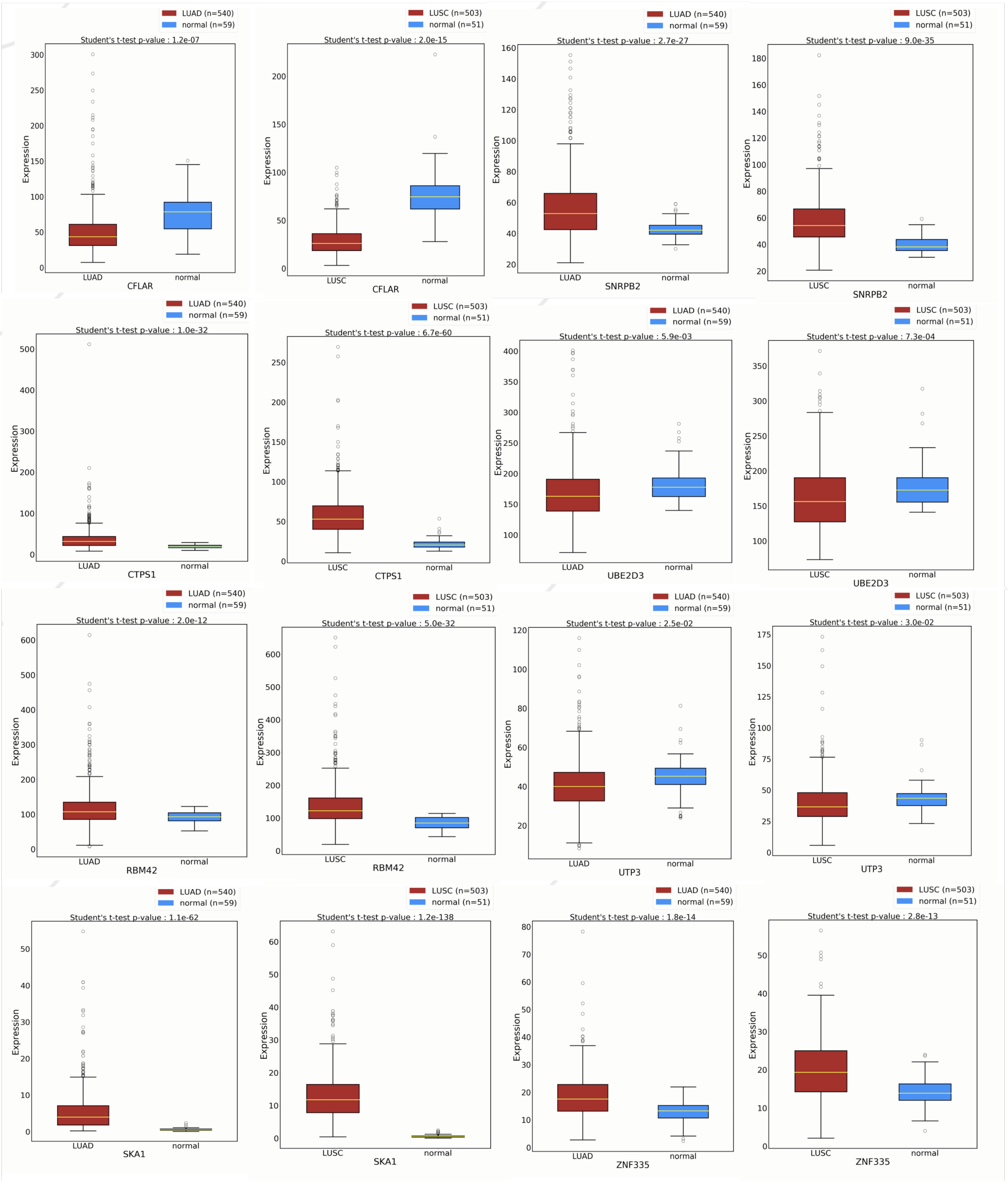

Fig D. Differential expression of NSCLC ucsEGs. The boxplots show the expression levels of the eight NSCLC-specific EGs in the two NSCLC subtypes, LUAD and LUSC, and normal samples, as collected in OncoDB. The significance of the average difference between the two populations was evaluated with a Student's t-test using the OncoDB platform tool for the differential expression analysis. The legends indicate the colours associated with the groups and the number of samples in brackets.

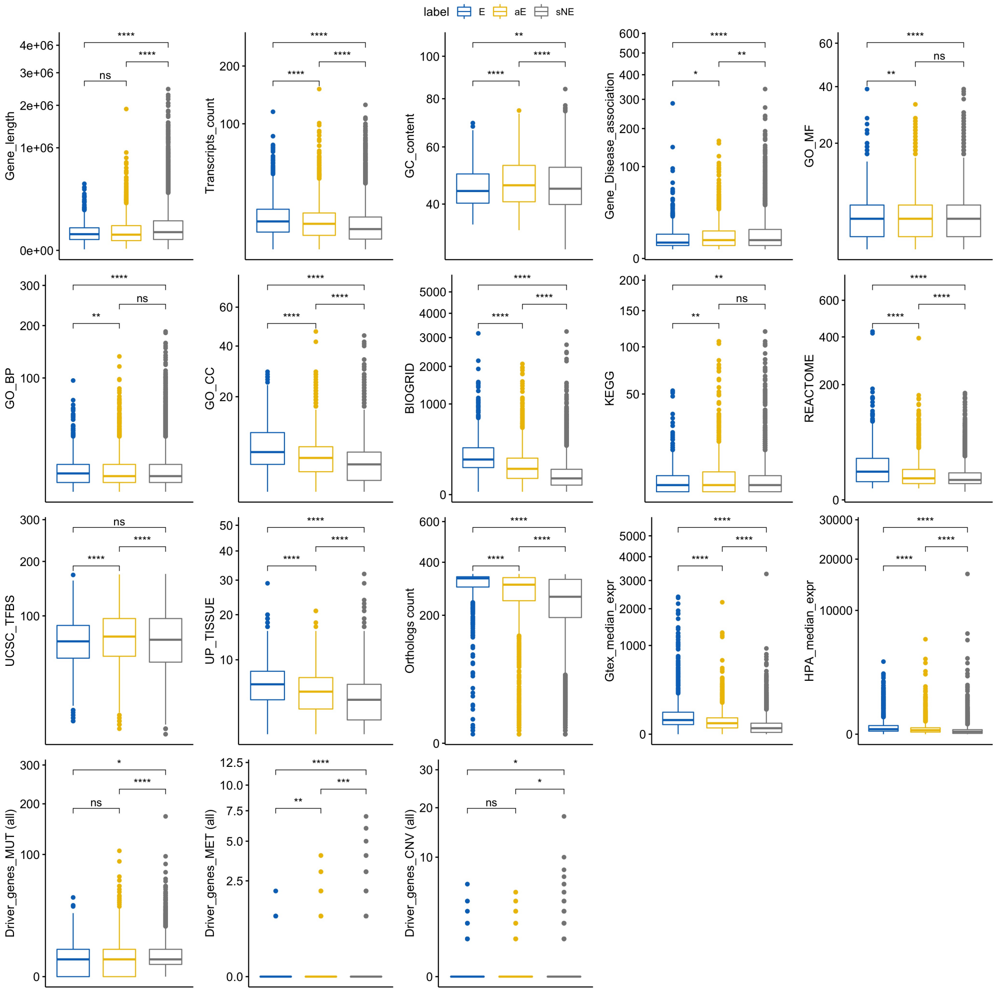

Fig E. Boxplots of the generic Human Bio attribute values for the E, aE, and sNE classes. The stars on the top indicate the significance of the Wilcoxon test for each pair of comparisons (**** ≤ 0.0001, *** ≤ 0.001, ** ≤ 0.01, * ≤ 0.05, ns = not significant). In favour of visualisation, the values have been signed-square-root transformed.

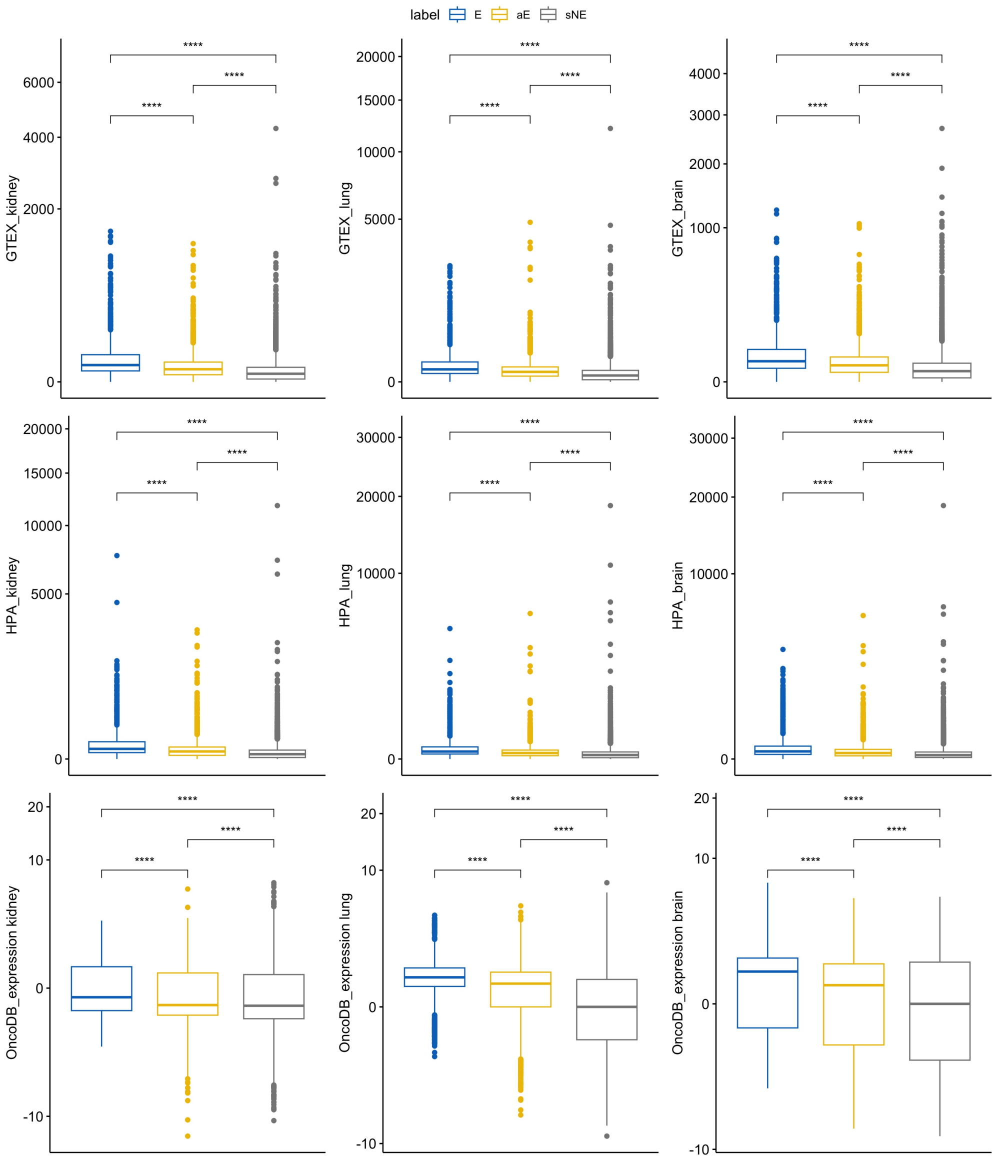

Fig F. Boxplots of the context-specific Bio attribute values of the three tissues investigated for the E, aE, and sNE classes. The stars on the top indicate the significance of the Wilcoxon test for each pair of comparisons (**** $\leq$ 0.0001, *** $\leq$ 0.001, ** $\leq$ 0.01, * $\leq$ 0.05, ns = not significant). The Driver genes attributes were not shown as having small ranges of values and poor statistics. In favour of visualisation, the values have been signed-square-root transformed.

(A)

Generic attribute	ns	*	**	***	****
Gene length	96	4	0	0	aE
Transcripts count	97	3	0	0	aE
GC content	96	2	2	0	aE
Gene-Disease association	95	5	aE	0	0
GO-MF	91;aE	8	1	0	0
GO-BP	93;aE	5	2	0	0
GO-CC	94	4	2	0	aE
BIOGRID	94	5	1	0	aE
KEGG	95;aE	3	2	0	0
REACTOME	95	5	0	aE	0
UCSC_TFBS	93	4	3	0	aE
UP_tissue	96	4	0	0	aE
Orthologs count	96	2	2	0	aE
Driver_genes_MUT (all)	94	6	0	aE	0
Driver_genes_MET (all)	94	4	2	aE	0
Driver_genes_CNV (all)	95	5;aE	0	0	0
**cs Attribute**	**ns**	*****	******	*******	********
GTEX_kidney	93	4	3	0	aE
GTEX_lung	92	8	0	0	aE
GTEX_brain	94	6	0	0	aE
OncoDB_expression kidney	94	4	2	0	aE
OncoDB_expression lung	96	2	1	1	aE
OncoDB_expression brain	95	5	0	0	aE
HPA_kidney	95	5	0	0	aE
HPA_lung	92	8	0	0	aE
HPA_brain	97	1	2	0	aE

(B)

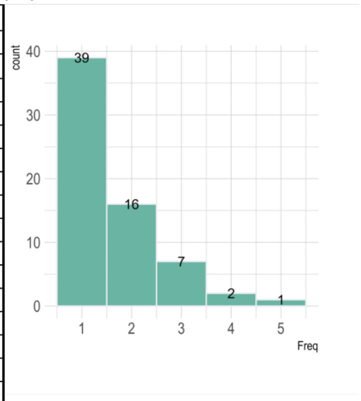

(C)

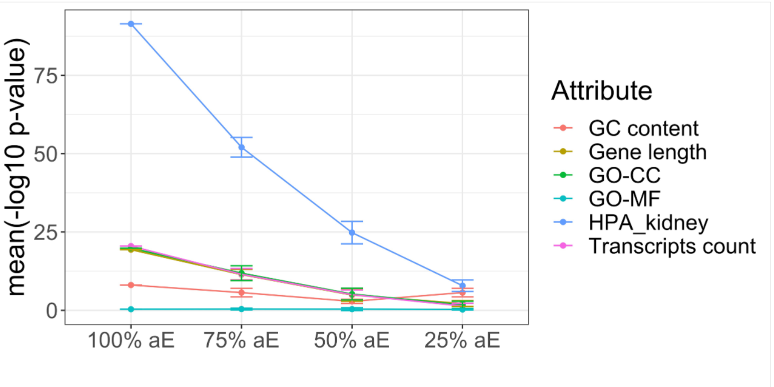

Fig G. Random extraction of the intermediate class. A) For each generic attribute (taken as an example from the Kidney dataset) and cs attributes from the three tissues, 100 random partitions of 3000 genes from the sNE groups have been extracted and compared to the rest of the sNE genes. For each tissue, the 100 partitions were fixed. Wilcoxon test was performed to evaluate the statistical significance (p-value) and verify whether the groups come from the same population for each pair of comparisons (**** $\leq$ 0.0001, *** $\leq$ 0.001, ** $\leq$ 0.01, * $\leq$ 0.05, ns = not significant). The table indicates the number of partitions for each attribute and for each significance level indicated in the column header. The level of significance given by comparing aE vs sNE, and indicated in Figs E and F, was also shown by the orange text "aE". B) The histogram shows the number of attributes (x-axis) for which the partitions are simultaneously significant. The count of partitions (y-axis) for each frequency is also shown on the bars. C) The line plot shows the mean of -log10(p-value) and the standard deviation from Wilcoxon tests between different percentages of aE mixed with sNE genes (to 3000 genes) obtained with 10 iterations and the rest of sNE genes for some attributes indicated in the legend.

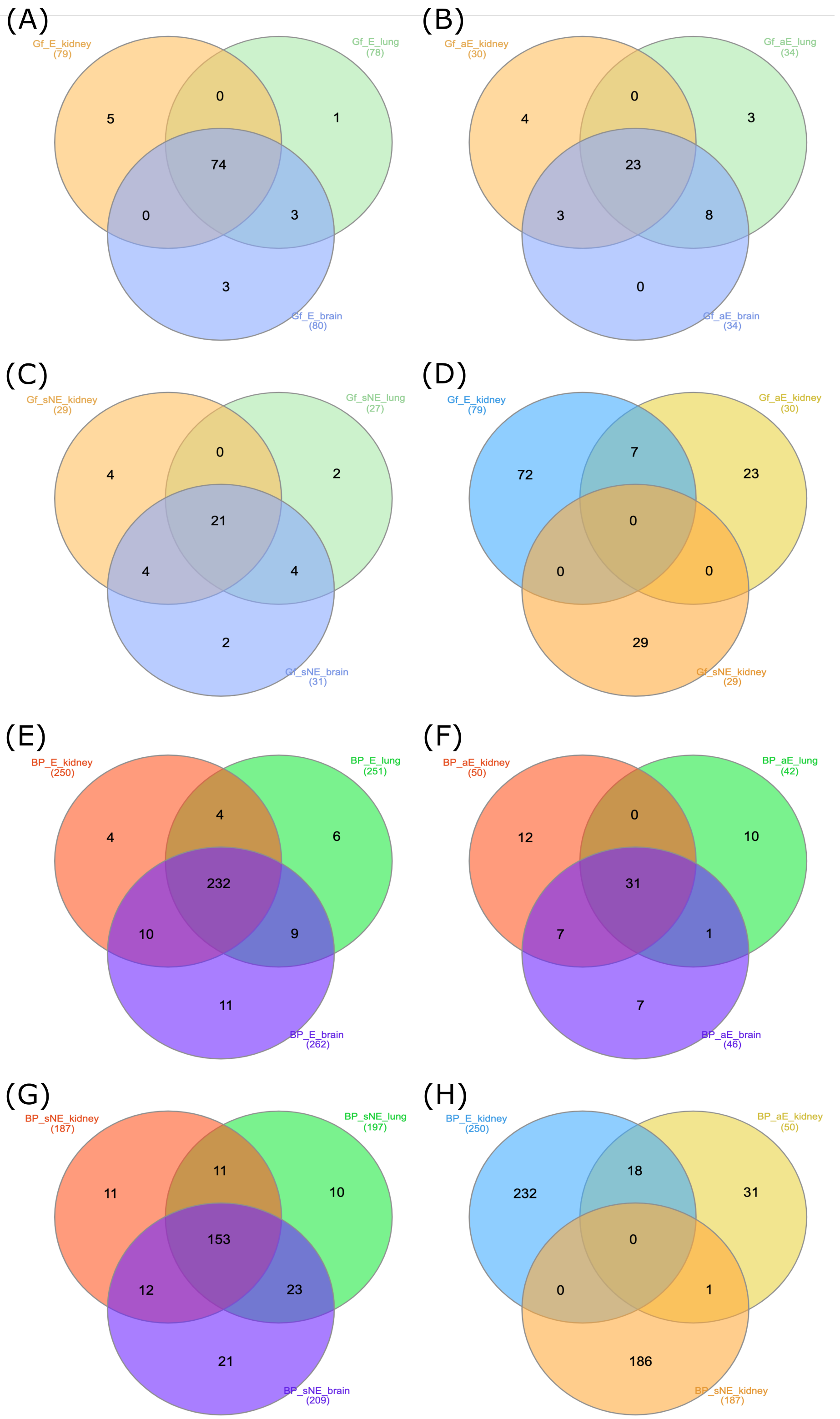

Fig H. Intersection of Gene Families and Biological Processes enrichment among E, aE and sNE genes. The Venn diagrams show the intersection of Gene Families (gf) and Gene-Ontology Biological Processes (BP) enriched by E, aE or sNE genes among the three tissue contexts under study (A-C; E-G), as well as the intersection of Gene Families (gf) and Gene-Ontology Biological Processes (BP) enriched by genes of the three classes in one context (here Kidney tissue as example) (D and H). The number of genes composing each set is shown in brackets.

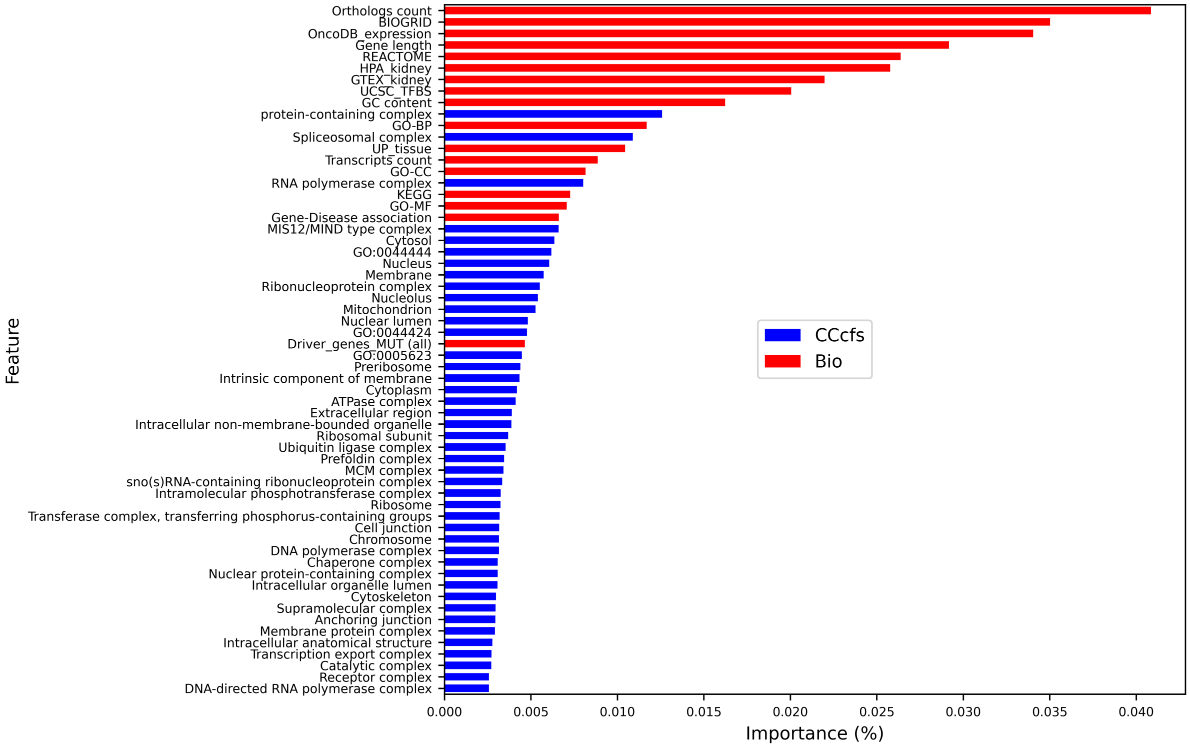

Fig I. Feature importance analysis. Bio+CCcfs attributes importance calculated by training a sveLGBM model on the entire dataset. The plot cuts-off feature with importance lower than 0.25 %.

Table A. Collected genomic, transcriptomic, epigenetic, functional and evolutionary features of genes. (cs) indicates the context-specific attributes.

**Category**	**Attribute**	**Description**	**Data Source**
Structure	Gene length<br>GC content<br>Transcripts count	Gene End (bp) - Gene Start (bp)<br>% of Guanosine + Citosine<br>No. of transcripts/gene	biomaRt R package v2.54 [2]
Expression	GTEX_* (cs)	Gene median expression in the context of interest	GTEX portal [3]
	UP_tissue	Count of annotated expression in tissues	DAVID [4]
	OncoDB_expression (cs)	Differential Gene Expression in cancer	OncoDB [5]
	HPA_* (cs)	Normalised transcript expression summarised per gene in the context of interest	HPA [6]
Function & Localisation	GO-MF<br>GO-BP<br>GO-CC<br>KEGG<br>REACTOME	No. of GO-MF annotations<br>No. of GO-BP annotations<br>No. of GO-CC annotations<br>No. of KEGG pathway annotations<br>No. of REACTOME pathway annotations	DAVID [4]
	CCcfs	Subcellular localisation confidence score	COMPARTMENTS [7]
Interaction	BIOGRID<br>UCSC_TFBS	No. of BIOGRID interactions annotations<br>Transcription factors binding sites prediction	DAVID [4]
Conservation	Orthologs count	No. of orthologous/gene	NCBI [8]
Association with Disease	Driver_genes_MUT (cs)<br>Driver_genes_CNV (cs)<br>Driver_genes_MET (cs)	No. of predictions as 'MUT driver' in cancer<br>No. of predictions as 'CNV driver' in cancer<br>No. of predictions as 'Methylation driver' in cancer	DriverDBv3 [9]
	Gene-Disease association	No. of associations with diseases	DisGeNet [10]

Table B. Comparison of classifiers on prediction in "E vs NE" problem in the Kidney case study. Ranking of methods is based on the Balanced Accuracy metric. All methods with "sve" prefix are our meta-learning model proposal with a different base classifier as member of the ensemble. All other methods are provided by the PyCaret library. All models where trained with Bio+CCcfs+N2V attributes of genes. CPU times are measured on Apple M2 with 16GB RAM.

Model	Accuracy	ROC-AUC	Sensitivity	Specificity	BA	TT (Sec)
sveLGBM	0.850100	0.951200	0.914800	0.845000	0.879900	14.608000
sveADA	0.856900	0.945400	0.901100	0.853500	0.877300	13.146000
sveET	0.866600	0.936400	0.852700	0.867600	0.860200	3.588000
sveRF	0.883200	0.938600	0.832000	0.887200	0.859600	3.008000
Random Forest Classifier	0.810200	0.903600	0.830800	0.808600	0.819700	0.916000
Extra Trees Classifier	0.826100	0.871100	0.761800	0.831100	0.796500	0.758000
Linear Discriminant Analysis	0.945500	0.931800	0.619100	0.970900	0.795000	6.512000
sveLDA	0.740800	0.856100	0.837800	0.733300	0.785500	5.074000
Logistic Regression	0.899400	0.842400	0.627200	0.920500	0.773900	1.572000
SVM - Linear Kernel	0.885200	0.827900	0.600700	0.907300	0.754000	19.138000
Ada Boost Classifier	0.943700	0.928900	0.492500	0.978700	0.735600	4.790000
Light Gradient Boosting Machine	0.947900	0.940600	0.474100	0.984700	0.729400	2.174000

Table C. sveLGBM tuning of parameters with Optuna library [11]. Optimiziation was carried out on “E vs NE” classification problem with a stratified 5-fold cross-validation with Bio+CCcfs+N2V features by maximising BA metric.

Trial no.	boosting_type	learning_rate	n_estimators	n_voters	BA
37	gbdt	0.094505	200	13	0.893151
15	gbdt	0.098300	140	10	0.891459
44	gbdt	0.076452	200	12	0.890954
43	gbdt	0.075168	200	12	0.890826
41	gbdt	0.078591	200	13	0.890602
33	gbdt	0.098020	180	13	0.890241
31	gbdt	0.059095	160	11	0.889936
34	gbdt	0.085756	200	13	0.889739
22	gbdt	0.063759	180	9	0.889298
30	gbdt	0.054934	160	12	0.889146
36	gbdt	0.076796	200	14	0.889028
23	gbdt	0.065602	160	9	0.888994
42	gbdt	0.076634	200	16	0.888759
40	gbdt	0.044127	180	10	0.888419
4	gbdt	0.088891	140	15	0.888175
39	gbdt	0.098960	140	14	0.887998
49	gbdt	0.057674	200	16	0.887826
11	gbdt	0.059871	180	15	0.886745
47	gbdt	0.049777	200	14	0.886566
29	gbdt	0.042902	180	9	0.886259
32	gbdt	0.052158	140	11	0.885557
...	...	...	...	...	...
5	gbdt	0.001175	100	7	0.500000

Table D. Classification performance metrics adopted in the experiments. They are defined in terms of the number of true positives (TP), true negatives (TN), false positives (FP), and false negatives (FN), where the first class in each binary task (e.g. class E in the “E vs NE” classification task) is assumed as the positive class.

Metric	Description	Formula
Accuracy	% of correctly classified samples	$\frac{\text{TP}+\text{TN}}{\text{TP}+\text{FP}+\text{FN}+\text{TN}}$
Specificity (TNR)	% of negative samples correctly classified	$\frac{\text{TN}}{\text{TN}+\text{FP}}$
Sensitivity (TPR)	% of positive samples correctly classified	$\frac{\text{TP}}{\text{TP}+\text{FN}}$
Balanced Accuracy (BA)	Average of Specificity and Sensitivity	$\frac{1}{2}(\text{Sensitivity} + \text{Specificity})$
ROC-AUC	Area Under the Receiver Operating Characteristic curve	$\int_0^1 \text{Sensitivity}(x)dx$, $x = 1 - \text{Specificity}$
CM	Confusion Matrix	$\begin{array}{c\|c} \text{TN} & \text{FP} \\ \hline \text{FN} & \text{TP} \end{array}$

Apart from the confusion matrix, all the metrics assume values in [0,1], except ROC-AUC, which ranges in [0.5,1]; higher values indicate better performance.

Table E. "E vs NE" classification performance based on HELP labelling. (A) Kidney, (B) Lung, (C) Brain tissues, and (D) Human. Averages and errors of metrics are obtained on fifty measurements related to ten times iterated 5-fold cross-validation. The averaged Confusion Matrix (CM) is also shown.

feature	Bio	N2V	CCcfs	Bio+CCcfs	Bio+CCcfs+N2V
**(A) Kidney**					
ROC-AUC	0.914±0.007	0.929±0.008	0.940±0.008	0.956±0.005	0.958±0.006
Accuracy	0.795±0.007	0.845±0.006	0.861±0.006	0.877±0.005	0.880±0.005
BA	0.832±0.010	0.854±0.013	0.867±0.012	0.887±0.010	0.892±0.009
Sensitivity	0.875±0.020	0.864±0.027	0.873±0.023	0.899±0.020	0.905±0.019
Specificity	0.789±0.007	0.843±0.007	0.861±0.006	0.876±0.005	0.878±0.005
CM (pred)	NE, E	NE, E	NE, E	NE, E	NE, E
CM (true NE)	12618.1, 3375.9	13486.2, 2507.8	13763.9, 2230.1	14003.7, 1990.3	14041.4, 1952.6
CM (true E)	155.3, 1086.7	169.0, 1073.0	157.7, 1084.3	125.2, 1116.8	117.8, 1124.2
**(B) Lung**					
ROC-AUC	0.918±0.006	0.931±0.008	0.941±0.006	0.957±0.005	0.959±0.005
Accuracy	0.800±0.007	0.852±0.005	0.845±0.014	0.878±0.005	0.882±0.005
BA	0.839±0.010	0.857±0.011	0.864±0.011	0.891±0.009	0.895±0.009
Sensitivity	0.884±0.019	0.863±0.022	0.885±0.031	0.905±0.017	0.910±0.018
Specificity	0.793±0.008	0.851±0.005	0.842±0.017	0.876±0.005	0.879±0.005
CM (pred)	NE, E	NE, E	NE, E	NE, E	NE, E
CM (true NE)	12701.7, 3308.3	13619.7, 2390.3	13486.1, 2523.9	14021.9, 1988.1	14078.9, 1931.1
CM (true E)	142.2, 1081.8	168.2, 1055.8	140.9, 1083.1	116.0, 1108.0	109.7, 1114.3
**(C) Brain**					
ROC-AUC	0.916±0.006	0.932±0.007	0.942±0.007	0.958±0.005	0.960±0.005
Accuracy	0.801±0.006	0.852±0.007	0.847±0.014	0.882±0.006	0.883±0.006
BA	0.833±0.008	0.859±0.011	0.866±0.011	0.893±0.008	0.895±0.008
Sensitivity	0.869±0.019	0.868±0.024	0.888±0.031	0.906±0.019	0.910±0.018
Specificity	0.796±0.007	0.850±0.008	0.844±0.017	0.880±0.007	0.881±0.007
CM (pred)	NE, E	NE, E	NE, E	NE, E	NE, E
CM (true NE)	12747.1, 3262.9	13612.7, 2397.3	13512.1, 2497.9	14094.4, 1915.6	14104.2, 1905.8
CM (true E)	161.4, 1072.6	162.7, 1071.3	137.7, 1096.3	116.2, 1117.8	111.1, 1122.9
**(D) Human**					
ROC-AUC	0.909±0.008	0.912±0.010	0.942±0.008	0.957±0.006	0.957±0.007
Accuracy	0.790±0.008	0.822±0.007	0.843±0.006	0.878±0.007	0.877±0.007
BA	0.825±0.011	0.831±0.012	0.867±0.011	0.889±0.011	0.888±0.013
Sensitivity	0.865±0.022	0.842±0.023	0.896±0.021	0.903±0.020	0.902±0.023
Specificity	0.784±0.009	0.820±0.007	0.839±0.007	0.876±0.007	0.875±0.007
CM (pred)	NE, E	NE, E	NE, E	NE, E	NE, E
CM (true NE)	12541.8, 3450.2	13113.1, 2878.9	13418.7, 2573.3	14003.3, 1988.7	13987.9, 2004.1
CM (true E)	167.7, 1074.3	196.0, 1046.0	129.3, 1112.7	120.8, 1121.2	121.4, 1120.6

Table F. Comparison of sveLGBM and CLEARER on OGEE+DEG labelling for the prediction of cEGs. *Hs Features* refer to the features collected for Homo Sapiens EGs prediction presented in the work [12]. sveLGBM hyperparameters: n_voters=16, learning_rate=0.1, n_estimators=200, boosting_type='gbdt'. CLEARER hyperparameter: RF n_estimators=500 as in [12].

method	sveLGBM (HELP)	RandomForest (CLEARER)
metric	Bio+CCcfs+N2V	Hs Features reduced by lasso
ROC-AUC	0.9728±0.0051	0.9682±0.0024
Accuracy	0.9111±0.0068	0.9625±0.0025
BA	0.9130±0.0144	0.7844±0.0123
Sensitivity	0.9152±0.0359	0.5834±0.0240
Specificity	0.9108±0.0090	0.9854±0.0019
CM (pred)	E, NE	E, NE
CM (true E)	755, 70	486, 347
CM (true NE)	1177, 12019	200, 13543

Table G. Comparison of sveLGBM, DeepHE and EPGAT predictions on HELP labelling for Kidney-, Lung-, Brain-specific EGs, and cEGs (Human). EPGAT running with PPI input and sublocalisation attributes. EPGAT hyper-parameters are optimised by using the provided tuning function. DeepHE running with DNA sequencing extracted features plus node2vec embedding 120-sized features extracted from the PPI. HELP running with Bio+CCcfs + N2V embedding 120-sized features extracted from the PPI.

	Kidney			Lung		
metric	EPGAT	DeepHE	sveLGBM	EPGAT	DeepHE	sveLGBM
AUC	0.902±0.007	0.921±0.016	0.957±0.006	0.913±0.009	0.916±0.021	0.958±0.005
Acc.	0.834±0.028	0.845±0.016	0.894±0.004	0.843±0.032	0.845±0.023	0.895±0.004
BA	0.824±0.012	0.845±0.016	0.890±0.009	0.832±0.014	0.845±0.023	0.892±0.010
Sens.	0.813±0.045	0.866±0.02	0.886±0.019	0.819±0.051	0.877±0.029	0.889±0.020
Spec.	0.835±0.033	0.824±0.024	0.894±0.004	0.845±0.037	0.812±0.028	0.895±0.005
	Brain			Human		
metric	EPGAT	DeepHE	sveLGBM	EPGAT	DeepHE	sveLGBM
AUC	0.908±0.012	0.921±0.009	0.959±0.005	0.880±0.017	0.91±0.02	0.957±0.007
Acc.	0.857±0.022	0.847±0.012	0.898±0.006	0.784±0.043	0.83±0.027	0.891±0.006
BA	0.833±0.008	0.847±0.012	0.894±0.009	0.798±0.020	0.83±0.027	0.886±0.013
Sens.	0.806±0.027	0.884±0.022	0.890±0.019	0.815±0.063	0.898±0.037	0.880±0.024
Spec.	0.861±0.026	0.811±0.024	0.898±0.006	0.781±0.050	0.762±0.047	0.892±0.007

Table H. Optimal hyper-parameters of sveLGBM, DeepHE and EPGAT methods used in comparison of Table G.

method	Kidney	Lung	Brain	Human
EPGAT	epochs=1000,<br>lr=0.005,<br>weight_decay=0.0005,<br>h_feats=[8,1],<br>heads=[8,1],<br>dropout=0.4	epochs=1000,<br>lr=0.005,<br>weight_decay=0.0005,<br>h_feats=[8,1],<br>heads=[8,1],<br>dropout=0.4	epochs=1000,<br>lr=0.00057,<br>weight_decay=0.000247,<br>h_feats=[32,8, 1],<br>heads=[8,4,1],<br>dropout=0.137	epochs=1000,<br>lr=0.0023,<br>weight_decay=0.000126,<br>h_feats=[64,1],<br>heads=[4,1],<br>dropout=0.34
DeepHE	epochs=50, batch_size=32, dropout=0.2, h_feats=[128,256,512], folding=1			
sveLGBM	n_voters=13, n_estimators=200, boosting_type=gbdt, learning_rate=0.1			

Table I. "E vs sNE", "E vs aE" and "aE vs sNE" classification performance based on HELP labelling. The case study is Kidney tissue using Bio+CCcfs+N2V features. Averages and errors of metrics are obtained on fifty measurements related to ten times iterated 5-fold cross-validation. The averaged Confusion Matrix (CM) is also shown.

problem	E vs sNE	E vs aE	aE vs sNE
ROC-AUC	0.973±0.004	0.895±0.009	0.751±0.010
Accuracy	0.915±0.005	0.797±0.012	0.713±0.007
BA	0.915±0.007	0.813±0.012	0.687±0.010
Sensitivity	0.916±0.016	0.849±0.021	0.644±0.019
Specificity	0.915±0.005	0.776±0.016	0.729±0.008
CM	true \ pred: sNE, E<br>sNE: 11790.0, 1096.0<br>E: 104.8, 1137.2	true \ pred: aE, E<br>aE: 2412.3, 695.7<br>E: 187.7, 1054.3	true \ pred: sNE, aE<br>sNE: 9396.4, 3489.6<br>aE: 1106.2, 2001.8

References

1. Szklarczyk D, Gable AL, Lyon D, Junge A, Wyder S, Huerta-Cepas J, et al. STRING v11: protein–protein association networks with increased coverage, supporting functional discovery in genome-wide experimental datasets. Nucleic acids research. 2019;47(D1):D607–D613. https://doi.org/10.1093/nar/gky1131 PMID: PMC6323986

2. Durinck S, Spellman PT, Birney E, Huber W. Mapping identifiers for the integration of genomic datasets with the R/Bioconductor package biomaRt. Nature Protocols. 2009;4:1184–1191. https://doi.org/10.1038/nprot.2009.97 PMID: PMC3159387

3. Ardlie KG, Deluca DS, Segrè AV, Sullivan TJ, Young TR Young, Gelfand ET, et al. The Genotype-Tissue Expression (GTEx) pilot analysis: Multitissue gene regulation in humans. Science. 2015;348(6235):648–660. https://doi.org/10.1126/science.1262110 PMID: PMC4547484

4. Sherman BT, Hao M, Qiu J, Jiao X, Baseler MW, Lane HC, Imamichi T, et al. DAVID: a web server for functional enrichment analysis and functional annotation of gene lists (2021 update). Nucleic Acids Res. 2022;50(W1):W216–W221. https://doi.org/10.1093/nar/gkac194 PMID: PMC9252805

5. Tang G, Cho M, Wang X. OncoDB: an interactive online database for analysis of gene expression and viral infection in cancer. Nucleic Acids Res. 2022;50(D1):D1334–D1339. https://doi.org/10.1093/nar/gkab970 PMID: PMC8728272

6. Uhlén M, Fagerberg L, Hallström BM, Lindskog C, Oksvold P, Mardinoglu A, et al. Tissue-based map of the human proteome. Science. 2015;347(6220). https://doi.org/10.1126/science.1260419 PMID: 25613900

7. Binder JX, Pletscher-Frankild S, Tsafou K, Stolte C, O'Donoghue SI, Schneider R, et al. COMPARTMENTS: unification and visualization of protein subcellular localization evidence. Database. 2014;2014. https://doi.org/10.1093/database/bau012 PMID: PMC3935310

8. Sayers EW, Bolton EE, Brister JR, Canese K, Chan J, Comeau DC, et al. Database resources of the National Center for Biotechnology Information in 2023. Nucleic Acids Res. 2023;51(D1):D29–D38. https://doi.org/10.1093/nar/gkaa892 PMID: PMC7778943

9. Liu SH, Shen PC, Chen CY, Hsu A-N, Cho Y-C, Lai Y-L, et al. DriverDBv3: a multi-omics database for cancer driver gene research. Nucleic Acids Res. 2020;48(D1):D863–D870. https://doi.org/10.1093/nar/gkz964 PMID: PMC7145679

10. Piñero J, Ramírez-Anguita JM, Saüch-Pitarch J, Ronzano F, Centeno E, Sanz F, et al. The DisGeNET knowledge platform for disease genomics: 2019 update. Nucleic Acids Res. 2020;48(D1):D845–D855. https://doi.org/10.1093/nar/gkz1021 PMID: PMC7145631

11. Akiba T, Sano S, Yanase T, Ohta T, Koyama M. Optuna: A Next-generation Hyperparameter Optimization Framework. In: Proceedings of the 25th ACM SIGKDD International Conference on Knowledge Discovery and Data Mining; 2019. https://doi.org/10.1145/3292500.3330701

12. Beder T, Aromolaran O, Dönitz J, Tapanelli S, Adedeji EO, Adebiyi E, et al. Identifying essential genes across eukaryotes by machine learning. NAR Genom Bioinform. 2021;3(4):lqab110. https://doi.org/10.1093/nargab/lqab110 PMID: PMC8634067
